# A Saturation Artifacts Inpainting Method Based on Two-Stage GAN for Fluorescence Microscope Images

**DOI:** 10.3390/mi15070928

**Published:** 2024-07-20

**Authors:** Jihong Liu, Fei Gao, Lvheng Zhang, Haixu Yang

**Affiliations:** 1 College of Information Science and Engineering, Northeastern University, Shenyang 110819, China; oliviagaofei@163.com (F.G.); lvhengzhang@163.com (L.Z.); 2 Department of Biomedical Engineering, Zhejiang University, Hangzhou 310027, China; yanghaixucn@163.com

**Keywords:** microscope image, deep learning, image inpainting, saturation artifacts

## Abstract

Fluorescence microscopic images of cells contain a large number of morphological features that are used as an unbiased source of quantitative information about cell status, through which researchers can extract quantitative information about cells and study the biological phenomena of cells through statistical and analytical analysis. As an important research object of phenotypic analysis, images have a great influence on the research results. Saturation artifacts present in the image result in a loss of grayscale information that does not reveal the true value of fluorescence intensity. From the perspective of data post-processing, we propose a two-stage cell image recovery model based on a generative adversarial network to solve the problem of phenotypic feature loss caused by saturation artifacts. The model is capable of restoring large areas of missing phenotypic features. In the experiment, we adopt the strategy of progressive restoration to improve the robustness of the training effect and add the contextual attention structure to enhance the stability of the restoration effect. We hope to use deep learning methods to mitigate the effects of saturation artifacts to reveal how chemical, genetic, and environmental factors affect cell state, providing an effective tool for studying the field of biological variability and improving image quality in analysis.

## 1. Introduction

Fluorescence microscopy is a microscopic technique that leverages the phenomenon of fluorescence for the observation of biological samples. It excites fluorescent dyes or fluorescent protein-tagged organisms or tissues with specific wavelengths of light, causing them to emit visible light. The resulting fluorescence microscope images contain many biologically relevant phenotypic features, enabling experimental characterization of gene expression.protein expression, and molecular interactions in a living cell [1]. The application of cell analysis based on fluorescence microscopy images is diverse, including identifying disease phenotypes, gene functions, and mechanisms of action, toxicity, or targets of drugs [2]. Analysis methods based on fluorescence microscope images, such as cell classification, segmentation, colocalization analysis, and morphological analysis, require high-quality microscopic images. However, the situation arises where proteins bind to an excessive amount of fluorescent dyelong exposure times, and inhomogeneous illumination [3], there are usually artifacts such as blurs, boundary shadows, and saturation artifacts that can interfere with the extraction of phenotypic features, thereby affecting the accuracy of the research findings. For example, uneven illumination increases the error detection and missed detection of yeast cell images by 35% via CellProfiler V2.2.0 [1], and saturation artifacts make the measurement of protein position invalid in colocalization location analysis. Therefore, investigating effective image processing and analysis methods to enhance the quality of fluorescence microscopic images and ensure the precision of phenotypic feature analysis holds significant importance for advancing the field of cell biology.

At present, the research on processing fluorescence image artifacts mainly focuses on inhomogeneous illumination, super-resolution reconstruction, and denoising. Smith et al. [4], Goswami et al. [5], and wang et al. [6] use prospective methods or retrospective methods to correct illumination between different fluorescence images and reduce abiotic structural differences between different images. These methods reduce abiotic structural differences between different images or remove the artifact noise from a single microscopic image. However, none of them can eliminate saturation artifacts in a single microscopic image. Saturation artifacts can be regarded as extreme illumination imbalances. Excessive exposure makes the artifact area blank, and a large area of biological structure information is missing. Often, these microphotographs with a large amount of missing biological structure information will be screened out in quantitative analysis experiments [7]. Among existing techniques for addressing saturation artifacts, approaches like those of Li et al. [8] and Hu et al. [9] predominantly employ a one-stage network to produce image characteristics. Yet, these networks face challenges in accurately reconstructing the intricate texture details present in the images.

Generative adversarial networks (GANs) were proposed by Goodfellow et al. [10] in 2014 as a tool for generating data. GANs and improved GAN algorithms have been widely used in image generation, image inpainting, and other fields by data-driven approaches in recent years and have excellent performance. Zhang et al. [11] used a GAN to provide an effective method for medical image data enhancement and forgery detection, effectively improving the accuracy and reliability of computer-aided diagnostic tasks. GANs have also had stunning success in the image processing of fluorescence microscopy. Chen et al. [12] used the GAN method to realize the super-resolution reconstruction of fluorescence microscope images, making the biological structure information stand out clearly from the artifact. In this paper, we propose a method to restore the missing biological structure information caused by saturation artifacts in each image. To our best knowledge, this is the first study to deal with this lost biological information. Belthangady et al. [13] showed that CNN-based techniques for inpainting missing image regions are well positioned to address the problem of losing information. Their work inspired us to believe that the deep learning method is a good way to solve the problem of losing biological information through saturation artifacts.

In this work, we further explore GAN-based methods to solve the problem of missing biological information due to saturation artifacts in fluorescence microscope images. The method is based on EdgeConnect GAN [14]; we call it Two-stage Cell image GAN (TC-GAN). To obtain more stable and credible inpainting results, the model adopts a two-step progressive repair method. In the first stage, the shape features of the cell and the context features between cells are restored using the proposed Edge-GAN. In the second part, the texture features and intensity-based features of the cell are restored using the proposed Content-GAN based on the edge information. We introduce contextual attention [15] architecture into the model to learn where to borrow or copy feature information from known background patches to generate missing patches. Using this model, images that have lost information can have their phenotypic features re-stored based on their existing phenotypic traits, thereby supplementing the scarce samples in morphological analysis experiments.

The structure of the paper is as follows. Section 2 describes the structure and loss function of the model. Section 3 introduces the data and processing methods. The image inpainting experiment and verification experiment are presented in detail in Section 4. The conclusion is made in Section 5.

## 2. Methodology

This chapter describes in detail the structure of the proposed fluorescence microscope cell image inpainting model and the loss function used.

### 2.1. Generative Adversarial Networks

GANs were proposed by Goodfellow et al. [10] in 2014 to generate signals with the same feature distribution as the training set. A typical GAN consists of a generator and a discriminator, where the generator tries to generate data that matches the distribution of the training set; the discriminator determines whether the input signal is the original signal or the signal generated by the generator.

However, the content generated based on a GAN usually has the problem of blurred edges of restored content or semantic mismatch between restored content and background content. We focus on the performance of the restoration results on four numerical features of a cell fluorescence microscopic image and use a two-stage GAN with a contextual attention layer to restore different feature contents.

### 2.2. Feature Restoration

The phenotypic features of cells provide the raw data for profiling. They can be extracted to quantitatively describe complex cell morphology phenotypes. Here, these phenotypic features can be separated into four categories [1,16]: (1) Shape features, which represent boundaries, size, or the shape of nuclei, cells, or other organelles. (2) Microenvironment and context features, including the distribution among cells and subcellular structures in the field of view. (3) Texture features, which describe the distribution of pixel intensity values within the cellular structure. These features can intuitively display the fluorescent protein structure of a single cell. (4) Intensity-based features, which are computed from actual intensity values on a single-cell basis. Intensity-based features are closely related to texture features. The intensity-based features dominate when analyzing a few pixels; as the number of distinguishable, discrete intensities increases within a small area, the texture features will dominate [17]. In fluorescence microscope images, saturation artifacts will cause sparse texture features.

We used a two-stage network (from Edge-GAN to Content-GAN) to restore the above four features from saturated artifacts. Edge-GAN is used to generate the phenotypes of shape and contextual features, including cell morphology and the direction of the cell’s centroid, thereby establishing the fundamental morphology of the cell phenotype. After determining this most basic and important information in saturation artifacts, the texture features can be further restored using the Content-GAN, and they are typically represented as the protein structure, organelle structure, and cytoplasm of the cell in our eyes. Contextual attention architecture [15] is added to the network structure to make the boundary and texture features of the patched area consistent with the surrounding cells in morphology.

### 2.3. Model Structure

**Figure 1:**
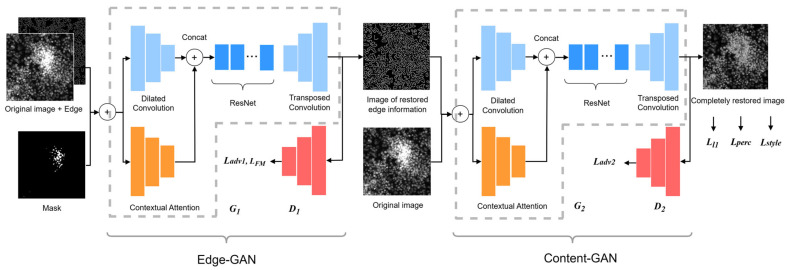
The modules of the proposed TC-GAN networks. $G_{1}$ and $D_{1}$ compose Edge-GAN; $G_{2}$ and $D_{2}$ compose Content-GAN.

The modules of our network are shown in Figure 1. The model is divided into two parts; we use Edge-GAN and Content-GAN, respectively, in these two parts. The Edge-GAN consists of a generator $G_{1}$ and discriminator $D_{1}$. The original grayscale image, the imaging mask, and the masked edge image obtained from the region of saturation artifacts using the Canny operator are the input of the Edge-GAN, generator $G_{1}$, and discriminator $D_{1}$. By learning the distribution of the features extracted from the input image, the Edge-GAN outputs the edge image. The Content-GAN consists of $G_{2}$ and $D_{2}$. The original grayscale image and edge grayscale image are the input of Content-GAN. By learning the texture features from the original image and the shape features from the edge image, the output is the restored image without saturation artifacts.

The generator of Edge-GAN, $G_{1}$, is composed of an encoder–decoder convolution architecture with a contextual attention architecture [15]. Specifically, the encoder–decoder architecture consists of the encoder, ResNet module, and decoder. The contextual attention architecture is parallel to the encoder architecture. The discriminator of Edge-GAN, $D_{1}$, follows the same architecture of 70 × 70 PatchGAN [18]; the detailed structures of $G_{1}$ and $D_{1}$ are shown in Table 1.

The architecture of the generator of Content-GAN, $G_{2}$, is the same as $G_{1}$, except all the spectral normalization is removed from $G_{2}$. And, the architecture of the discriminator of Content-GAN, $D_{2}$, is the same as $D_{1}$. $D_{2}$ is used to judge whether the semantic information of the content generated by $G_{2}$ is reasonable or not.

### 2.4. Contextual Attention

Contextual attention architecture is proposed by Yu et al. [13] to learn where to borrow or copy feature information from known background patches to generate missing patches. Its detailed structure is shown in the contextual attention architecture of Table 1. We use the contextual attention layer to accelerate the convergence rate of model training and enhance the semantic rationality of the generating region. The similarity of a patch centered in the patch to be restored $f_{x,y}$ and the background patch $b_{x^{\prime },y^{\prime }}$ is defined as

(1) $$S_{x,y,x^{\prime },y^{\prime }}=\langle \frac{f_{x,y}}{\left\|f_{x,y}\right\|},\frac{b_{x^{\prime },y^{\prime }}}{\left\|b_{x^{\prime },y^{\prime }}\right\|}\rangle$$

According to the calculated similarity score $S_{x,y,x^{\prime },y^{\prime }}$, the contextual attention layer can learn which part of the background features should be used from the repaired texture information.

### 2.5. Edge-GAN Loss Function

The Edge-GAN is trained with adversarial loss and feature-matching loss [19] as

(2) $$\underset{G_{1}}{\min}\underset{D_{1}}{\max}L_{G_{1}}=\underset{G_{1}}{\min}\left(\lambda_{adv1}\underset{D_{1}}{\max}\left(L_{adv1}\right)+\lambda_{FM}L_{FM}\right)$$

where $L_{adv1}$ is adversarial loss, $L_{FM}$ is feature-matching loss, and $\lambda_{adv1}$ and $\lambda_{FM}$ are regularization parameters. The adversarial loss $L_{adv1}$ is defined as

(3) $$L_{adv1}=E_{\left(E,I\right)}\left[\log D_{1}\left(E,I\right)\right]+E_{I}\log\left[1-D_{1}\left({\tilde{Z}}_{pred},I\right)\right]$$

where I is the ground truth images, E is the edge map of I, and ${\tilde{Z}}_{pred}$ is the predicted edge map for the masked region.

The feature-matching loss $L_{FM}$ extracts the middle feature layer of the discriminator for comparison. The $L_{FM}$ is defined as

(4) $$L_{FM}=E\left[\sum_{i=1}^{L}\frac{1}{N_{i}}{\left\|D_{1}^{\left(i\right)}\left(E\right)-D_{1}^{\left(i\right)}\left({\tilde{Z}}_{pred}\right)\right\|}_{1}\right]$$

where i means the number of feature layers, L is the final layer of $D_{1}$, $N_{i}$ is the number of elements in ith layer, and $D_{1}^{\left(i\right)}$ is the ith layer of $D_{1}$.

In our experiments, $\lambda_{adv1}=1$ and $\lambda_{FM}=10$.

### 2.6. Content-GAN Loss Function

The Content-GAN is trained by four losses. The overall loss function is to $\underset{G_{2}}{\min}\underset{D_{2}}{\max}L_{G_{1}}$, which is defined as

(5) $$\underset{G_{2}}{\min}\underset{D_{2}}{\max}L_{G_{1}}=\underset{G_{2}}{\min}\left(\lambda_{l_{1}}L_{l_{1}}+\lambda_{adv2}\underset{D_{2}}{\max}\left(L_{adv2}\right)+\lambda_{p}L_{prec}+\lambda_{s}L_{style}\right)$$

where $L_{l_{1}}$ is $l_{1}$ loss, $L_{adv2}$ is adversarial loss, $L_{perc}$ is perceptual loss [20], and $L_{style}$ is style loss [21]. $L_{l_{1}}$, $L_{adv2}$, $\lambda_{p}$, and $\lambda_{s}$ are regularization parameters.

Adversarial loss, $L_{adv2}$, is defined as

(6) $$L_{adv2}=E_{\left(I,{\tilde{Z}}_{comp}\right)}\left[\log D_{2}\left(I,{\tilde{Z}}_{comp}\right)\right]+E_{{\tilde{Z}}_{comp}}\log\left[1-D_{2}\left(Z_{pred},{\tilde{Z}}_{comp}\right)\right]$$

where the composite edge map ${\tilde{Z}}_{comp}=E⊙\left(1-M\right)+{\tilde{Z}}_{pred}⊙M$ and the inpainting color image $Z_{pred}=G_{2}\left(Z,{\tilde{Z}}_{comp}\right)$.

The perceptual loss is similar to the feature-matching loss, which extracts the middle feature layer for comparison in $D_{2}$. Using perceptual loss can avoid generating final content that is the same as the input image, as long as the abstract features are the same. This is defined as

(7) $$L_{perc}=E\left[\sum_{i=1}\frac{1}{N_{i}}{\left\|\phi_{i}\left(I\right)-\phi_{i}\left(Z_{pred}\right)\right\|}_{1}\right]$$

where $\phi_{i}$ is the activated feature map of the ith layer of $D_{2}$. Here, we use the VGG-19 pre-trained parameter on the ImageNet dataset [22] to be the parameter of $\phi_{i}$.

The style loss is used to punish the non-intensity affine transformation and reduce the distortion of cell morphological transformation. It is defined as

(8) $$L_{style}=E_{j}\left[{\left\|G_{j}^{\phi }\left(I⊙\bar{M}+Z_{pred}⊙M\right)-G_{j}^{\phi }\left(Z\right)\right\|}_{1}\right]$$

where $G_{j}^{\phi }$ is a Gram matrix constructed of $C_{j}\times C_{j}$ from feature maps $\phi_{j}$, $\bar{M}=1-M$.

## 3. Data and Processing

### 3.1. Data of Fluorescence Microscope Image

The data used in this study are obtained from the training set of RxRx1 in the NeurIPS 2019 competition track (https://www.kaggle.com/c/recursion-cellular-image-classification/data, accessed on 23 November 2021). This database contains fluorescence microscope images of cells collected from each well plate in high-throughput screening (HTS).

The original RxRx1 data contain four types of cells (HUVEC, RPE, HepG2, and U2OS). There are 1108 different small interference RNAs (siRNAs) introduced into four types of cells to create distinct genetic conditions. The experiment uses a modified cell painting staining protocol that uses six different stains to adhere to different parts of the cell. The stains fluoresce at different wavelengths and are, therefore, captured by different imaging channels. Thus, there are six channels per imaging site in a well.

**Figure 2:**
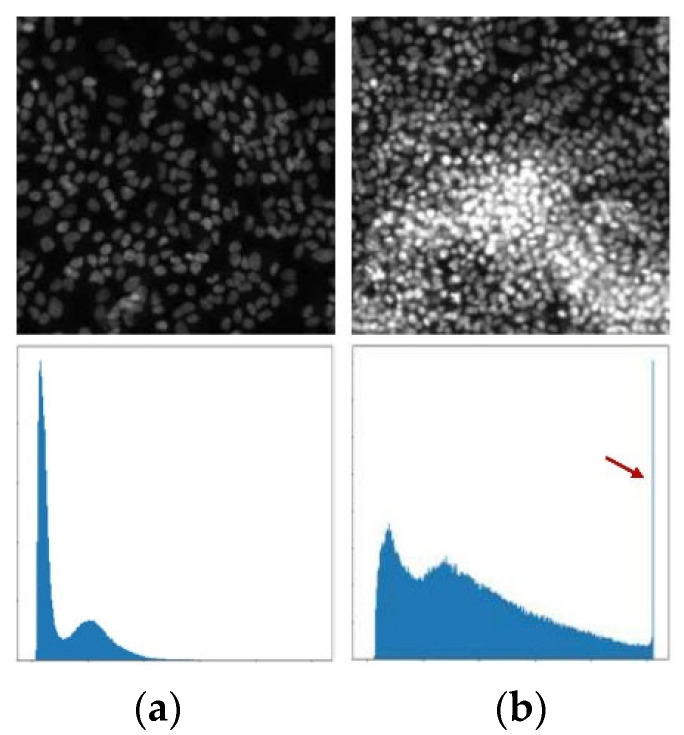
Examples of (**a**) normal images and (**b**) problematic images with saturated pixels with their histogram. (**a**) shows the images with normal grayscale value distribution. The red arrow in (**b**) shows a large number of saturated pixels caused by saturation artifacts. Images with saturated pixels have more pixels with a value of 255, which means losing a lot of information.

Different types of cell information are reflected in the morphological differences in fluorescence microscope images. The morphological analysis of cells is usually based on these morphological features. The most significant influences on the features of morphological differences are the saturation artifacts, which are shown in Figure 2.

In the RxRx1 dataset, different strategies are adopted to select data that are significantly affected by saturation artifacts and data that are free from saturation artifacts and rich in edge information. Saturation artifacts in the images are characterized by clusters of saturated pixels with pixel values reaching 255. When there is a high concentration of saturated pixels gathering in the same area, it can lead to large areas of structural loss in the image. This study screens the data based on the proportion of saturated pixels in the entire image, selecting images where the mean and standard deviation of the overall pixel values are both greater than 20, to identify the data significantly affected by saturation artifacts. Data selected for being devoid of saturation artifacts and possessing abundant edge information meet two specific criteria: firstly, there should be no pixels with a value of 255 within the image, and secondly, the image must exhibit a discrete entropy value exceeding 5. Discrete entropy is defined as

(9) $$H=-\sum_{i}P_{i}\log_{2}P_{i}$$

where $P_{i}$ is the probability of the occurrence of a pixel with a grayscale value of i in an image.

### 3.2. Training Set Preparation

The data used in this study were divided into three groups (T1, SET1, and SET2). The data in T1 were selected from RxRx1 and did not contain saturation artifacts and were morphologically rich. This ensures that the trained algorithm can fill with rich textures. SET1 and SET2 are used to evaluate the validity of the restored feature. SET1 includes 100 images without saturation artifacts selected from original RxRx1 data, 100 masked images that are masked in 20% of the area to simulate the saturated artifact, and 100 images restored via TC-GAN. SET2 includes five images affected by saturation artifacts selected from original RxRx1 data and five images restored using TC-GAN.

## 4. Training Strategy and Analysis

In this section, we first introduce the experimental progressive training strategy and its ablation experiment results, and the training process and experimental results of TC-GAN are introduced. We used peak signal-to-noise ratio (PSNR), structural similarity index (SSIM), and Fréchet Inception Distance (FID) to evaluate the validity of the restoration results.

### 4.1. Training Strategy

It is challenging to restore the phenotypic features directly, especially the shape features and context features of cells in a large area of saturation artifacts. We use the method of progressive generation in the process of Edge-GAN training. And, by using the results of Edge-GAN, Content-GAN can restore apparent texture features.

**Figure 3:**
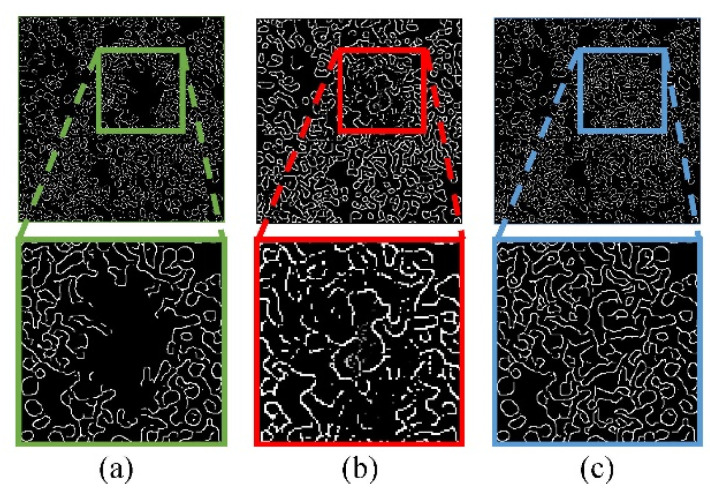
The results of progressive restoration. (**a**) Shape features extracted from the image when there are saturated artifacts, (**b**) shape features restored in the first stage, (**c**) shape features restored in the second stage.

Specifically, the Edge-GAN trains on low-resolution images for pre-training, and then, we use transfer learning based on pre-training to train Edge-GAN on high-resolution images. The results of shape feature and context feature restoration in stages are shown in Figure 3. As it shows, the phenotypic features between cells are gradually restored.

**Figure 4:**
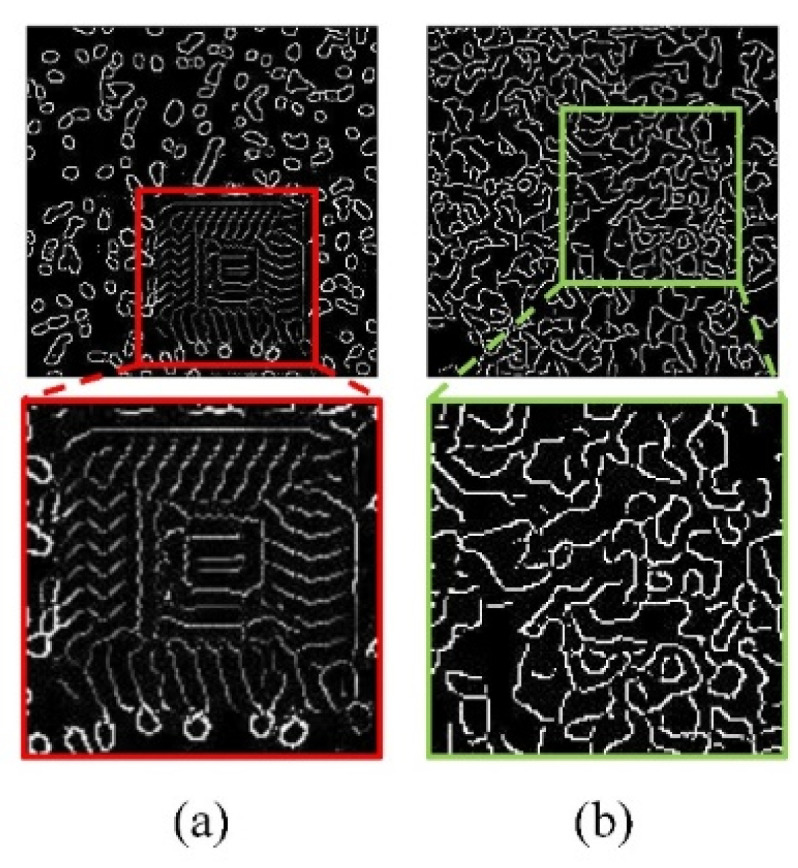
The results of progressive restoration ablation experiments. (**a**) is the training result of direct restoration on high-resolution images, and (**b**) is the training result obtained by gradually training from low-resolution images to high-resolution images.

In this study, an ablation experiment was carried out, and the experimental results of progressive restoration and no progressive restoration were compared; the restoration results shown in Figure 4 show the restoration results of missing edge information at the 50,000th step in Edge-GAN.

### 4.2. Model Training and Result

The restoration TC-GAN models are trained using T1 data, which have rich phenotypic features without saturation artifacts. Images in the training set, imaging mask, and the masked edge consist of the input of TC-GAN. And, the final output of TC-GAN is the restored image without saturation artifacts. Here, the training process for two-stage TC-GAN can be described as follows:

1. Low-resolution original image, imaging mask, and masked edge consist of the input of Edge-GAN;
2. Generator $G_{1}$ outputs the edge image as the output of Edge-GAN;
3. Compute the $L_{G_{1}}$ and the gradient of $G_{1}$ and $D_{1}$ and return to step 1 until the training of Edge-GAN finishes;
4. Replace the low-resolution image with the high-resolution image and return to step 1 until the training of Edge-GAN finishes;
5. The edge image and the original image consist of the input of Content-GAN;
6. Generator $G_{2}$ outputs the restored image as the output of Content-GAN;
7. Compute the $L_{G_{2}}$ and the gradient of $G_{2}$ and $D_{2}$ and return to step 5 until the training of Content-GAN finishes.

For Edge-GAN, the optimizer is Adam [23] with a learning rate of α= 0.0001, $\beta_{1}=$ 0, $\beta_{2}=$ 0.9. The total training iteration is 1,000,000. For Content-GAN, the optimizer is the same as Edge-GAN, and the training iteration is 200,000.

**Figure 5:**
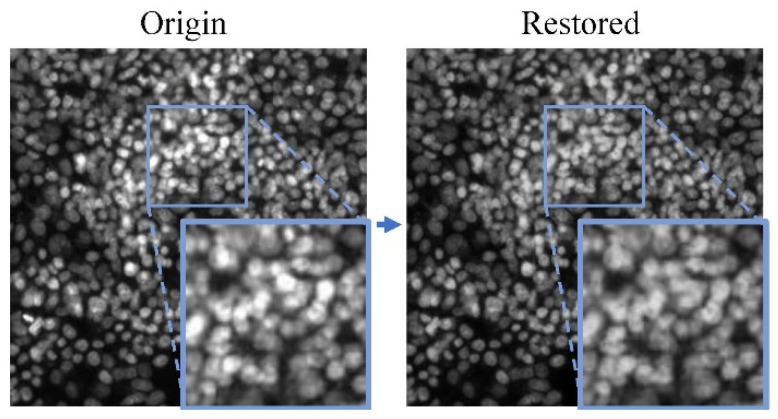
One demo of images with saturation artifacts restored using TC-GAN.

The result of the two-stage restoration of the network is shown in Figure 5. As shown, the restored image fills the lost phenotypic features in the original saturation artifacts area. Additional examples have been provided in Section 4.3.3.

### 4.3. Evaluation of Validity

#### 4.3.1. Evaluation Indicators

We use several quantitative statistical features to verify the effectiveness of the method. The peak signal-to-noise ratio (PSNR), the structural similarity index (SSIM) [24], and Fréchet Inception Distance (FID) [25] are used to evaluate the quality of generation features quantitatively.

PSNR evaluates the quality of the generated features compared with the original features. The higher the PSNR, the smaller the distortion of the generated features.

SSIM is an index to measure the similarity of features in two images. The closer the SSIM is to 1, the closer the patched features are to the original cell.

#### 4.3.2. Evaluation Methods

The PSNR, SSIM, and FID of SET1 are calculated to evaluate the validity of generation features. We calculate the PSNR, SSIM, and FID between the 100 original images without saturation artifacts and the 100 masked images after they are artificially covered in SET1 to obtain the data of the mask group. Then, we calculate the PSNR, SSIM, and FID between the 100 original images and the 100 restored images after being covered in SET1 to obtain the restoration group data. Calculation results of the mask group and restoration group can show the image quality before and after restoration. In addition, they can reflect the similarity between the restoration area and the original cell.

To visually verify the effectiveness of the restoration results, this study selected five images that were significantly affected by saturation artifacts and performed restoration on them, resulting in five restored images. These images constitute the validation set known as SET2.

#### 4.3.3. Result

**Figure 6:**
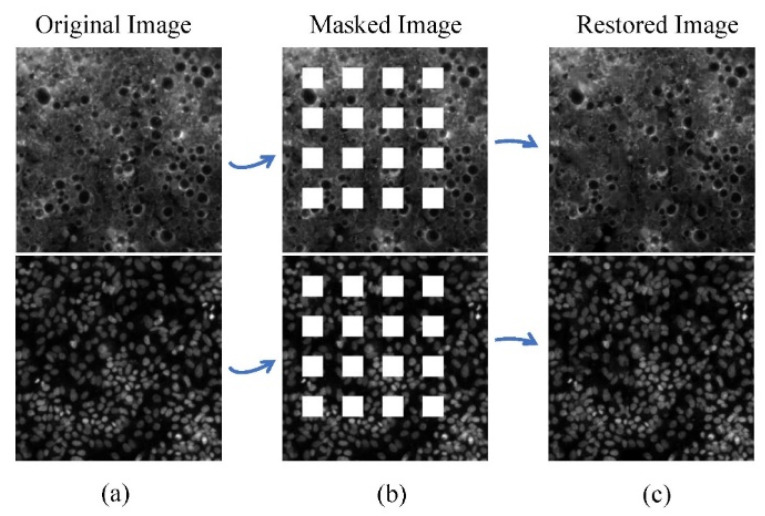
Two demos of (**a**) original images, (**b**) masked images, and (**c**) restored images of SET1. The (**b**) masked images lose some of their original morphological features in (**a**), and these missing morphological features are restored in the (**c**) restored images.

We use PSNR, SSIM, and FID to evaluate the validity of the generation features in SET1. The index difference between the restored image and the masked image is shown in Table 2. The PSNR and SSIM of the restored image are higher than those of the masked image. This means the restored phenotypic features can effectively fill the gap of saturation artifacts and make the restored image closer to the original image than the masked image. The FID of the restored image is lower than that of the masked image, which means the similarity between the restored image and the original image is higher than that between the masked image and the original image. Two examples of the original image, the masked image, and the restored image are shown in Figure 6.

**Figure 7:**
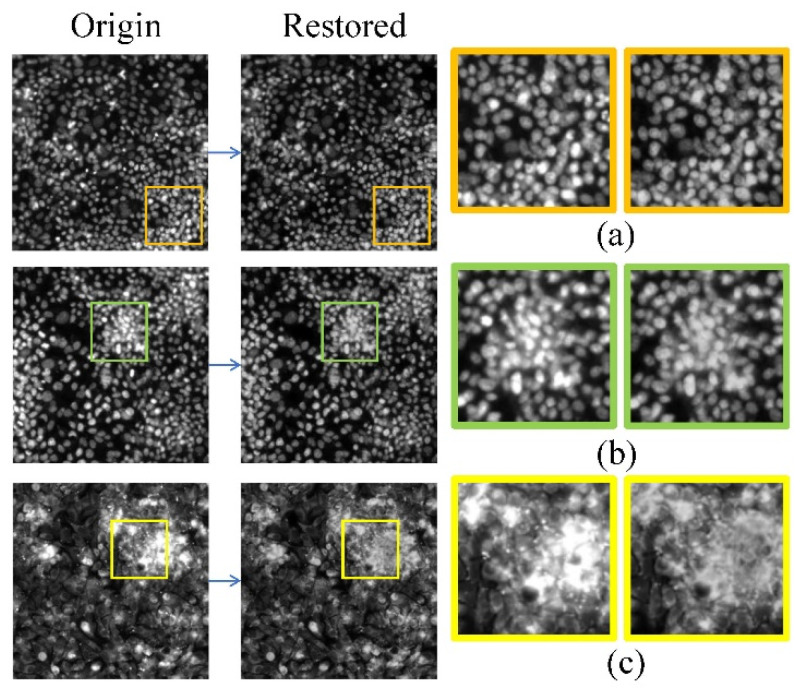
The patching result of all images in SET2. The images before and after restorations are given in (**a**–**c**).

The results of image restoration in SET2 are shown in detail in Figure 7, which shows the results of image restoration for images with real saturation artifacts. The large area of saturation artifacts in the original image no longer exists in the restored image. The context features between cells in the artifact areas and the intracellular texture features are restored using TC-GAN.

## 5. Conclusions

This paper introduces the TC-GAN model, a two-stage phenotypic feature restoration approach addressing saturation artifacts in fluorescence microscopy images. The model separately restores the shape and texture features of cells. Through ablation studies and quantitative and qualitative experiments, the effectiveness of the network under progressive training is validated. The results demonstrate the model’s practical significance and its potential to enhance the qualitative and quantitative analysis of cell fluorescence microscopy images.

## Figures and Tables

**Table 1:** The structures of $G_{1}$ and $D_{1}$.

		Input	Filter	Channel/Stride/Padding	Act	Output
$G_{1}$	Encoder Architecture	X	Conv 7 × 7	64/1/0	S/I/ReLU	
			Conv 4 × 4	128/2/1	S/I/ReLU	
			Conv 4 × 4	256/2/1	S/I/ReLU	Encoder $X_{1}$
	Contextual Attention Architecture	X	Conv 5×5	32/1/2	ELU	
			Conv 3 × 3	32/2/1	ELU	
			Conv 3 × 3	64/1/1	ELU	
			Conv 3 × 3	128/2/1	ELU	
			Conv 3 × 3	128/1/1	ELU	
			Conv 3 × 3	128/1/1	ReLU	
			Contextual Attention Layer			
			Conv 3 × 3	128/1/1	ELU	
			Conv 3 × 3	128/1/1	ELU	Feature $X_{2}$
	ResNet Architecture	$X_{1}+X_{2}$	Conv 3 × 3	384/1/0	S/I/ReLU	
			Conv 3 × 3	384/1/0	S/I	
			(ResNet Block × 8)			Feature $X_{3}$
	Decoder Architecture	$X_{3}$	TransposeConv 4 × 4	128/2/1	S/I/ReLU	
			TransposeConv 4 × 4	64/2/1	S/I/ReLU	
			Conv 7 × 7	1/1/0	Sigmoid	Y
$D_{1}$	Encoder Architecture	Y	Conv 4 × 4	64/2/1	S/LReLU	
			Conv 4 × 4	128/2/1	S/LReLU	
			Conv 4 × 4	256/2/1	S/LReLU	
			Conv 4 × 4	512/1/1	S/LReLU	
			Conv 4 × 4	1/1/1	LReLU/Sigmoid	1 × 32 × 32

Conv = Convolution filter, S = Spectral normalization, I = Instance normalization, LReLU = LeakyReLU. X is the input image of $G_{1}$, which consists of three channels: the original grayscale image, the imaging mask, and the masked edge image. $X_{1}$, $X_{2}$, and $X_{3}$ are the feature maps calculated using the middle layer. Y is the input image of $D_{1}$, which is the output image of $G_{1}$. The structure of $G_{2}$ is almost the same as $G_{1},$ except the ResNet of $G_{2}$ has 4 layers instead of 8 layers, and the loss function of $G_{2}$ is different from $G_{1}$. The structure of $D_{2}$ is the same as $D_{1}$.

**Table 2:** Indices of masked and restored images.

Dataset	PSNR	SSIM	FID
image with mask	9.101	0.725	609.154
image be repaired	25.948	0.854	50.345

## Data Availability

The original contributions presented in the study are included in the article, further inquiries can be directed to the corresponding author.
